# Promoter strength and position govern promoter competition

**DOI:** 10.1101/2025.05.06.652547

**Published:** 2025-05-07

**Authors:** Mervenaz Koska, Tomek Swigut, Alistair Nicol Boettiger, Joanna Wysocka

**Affiliations:** 1 Department of Chemical and Systems Biology, Stanford University, Stanford, CA 94305, USA; 2 Department of Developmental Biology, Stanford University, Stanford, CA 94305, USA; 3 Institute for Stem Cell Biology and Regenerative Medicine, Stanford University, Stanford, CA 94305, USA; 4 Howard Hughes Medical Institute, Stanford University, Stanford, CA 94305, USA; 5 Lead contact

## Abstract

Competition between promoters within a shared regulatory landscape has been implicated in development and disease, but the determinants of promoter competition have not been systematically studied. Here, we use a synthetic platform to introduce diverse promoters at defined genomic sites within the *Sox2* locus and measure how these inserted promoters attenuate activity of the endogenous promoter. We find that reduction in endogenous *Sox2* transcription is correlated with the strength of the inserted promoter. Transcription from the inserted promoter is required for competition, with longer transcript resulting in more competition. Furthermore, competition is dependent on the location of the inserted promoter, but independent of cohesin mediated loop extrusion. Lastly, we encounter silencing of the *de novo* inserted promoter by HUSH, which counteracts competition. Together, our work uncovers the rules governing promoter competition, highlights its impact on tuning gene expression levels, and suggests a role for RNA in mediating this process.

## INTRODUCTION

Gene expression is coordinated through a complex functional interplay between multiple classes of cis-regulatory elements, such as enhancers, promoters, boundary elements and faciliators^1-4^. Typically, multiple enhancers and promoters are present within a given regulatory domain, raising a question of how these cis-regulatory elements influence each other’s activity^5,6^. One phenomenon that has been previously described in the literature is ‘promoter competition’, referring to a mechanism whereby a promoter can dampen the transcriptional output of another promoter located in cis^7-9^. The prevalent model for explaining this phenomenon is the competition of the promoters for the shared enhancers within the same regulatory domain. A canonical example of such competition comes from the beta-globin locus, where the fetal and adult globin promoters compete for the distal locus control region^7,10^. In this system, activation of the adult b-globin promoters facilitates shutdown of the fetal globin promoter through competition^7^. On the contrary, disrupting any of the adult b-globin promoters redirects their cognate b-globin enhancers to fetal globin promoter and reactivates its expression^11^.

Although promoter competition likely occurs at many (if not most) endogenous loci in diverse biological contexts during development and in disease, such effects can be challenging to reveal without mutating a given promoter and assaying the impact of such perturbation on the activity of other promoters located in cis. The effects of promoter competition are more noticeable when a *de novo* promoter is introduced into a genomic region and causes changes in activity of the endogenous promoters nearby. An interesting example of this is the disease-causing single nucleotide variant in the human alpha-globin locus, which generates a transcription factor binding site that allows for promoter activity^12^. This *de novo* promoter disrupts the communication between the a-globin super enhancer cluster and its cognate promoters, thus leading to a reduction in a-globin gene expression and causing a-thalassemia^12^. Similarly, insertion of an active retrotransposon can result in promoter competition leading to the downregulation of endogenous promoter activity, as exemplified by the retroelement Idefix at the *white* locus in Drosophila ^13^.

While these compelling examples suggest a widespread impact of the interplay between the promoters on gene expression, the rules governing promoter competition have not been systematically studied. To address this gap in knowledge, we set out to explore how an existing cis-regulatory network responds to the introduction of a new promoter. To this end, we turned to the well-studied *Sox2* locus in mouse embryonic stem cells (mESC), where the majority of *Sox2* expression is controlled by a single super enhancer (SE) that lies ~110 kb away from the promoter^14,15^. At this locus, we introduced a set of diverse promoters at defined landing pad sites and assayed gene expression driven by both the inserted promoter and the endogenous *Sox2* promoter. We found that the ability of the inserted promoter to attenuate activity of the native *Sox2* promoter is inversely correlated with its strength and influenced by its orientation. Moreover, the competition is also dependent on the position of the inserted promoter in relation to the endogenous promoter and the SE, despite the inserted promoter receiving comparable enhancer input into activity regardless of its position. Remarkably, promoter competition is counteracted by silencing of the inserted promoter by the Human Silencing Hub (HUSH), a repressive complex that co-transcriptionally targets non-self DNA such as transgenes or transposable elements^16,17^. Consequently, removal of HUSH exacerbates promoter competition. We further show that although cohesin accumulates at the ectopic promoter, it does not drive competition. Instead, competition requires transcription downstream from the transcription start site (TSS) of the inserted promoter. The extent of the transcriptional readthrough (and hence, transcript length) is influenced by the insert orientation and in turn impacts competition, explaining the observed directionality effect. Altogether, our work shows that promoter strength and position – and the related level and length of the resulting transcripts – are key determinants of promoter competition.

## RESULTS

### A synthetic platform to study determinants of promoter competition

**Extended Data Fig. 1 –:**
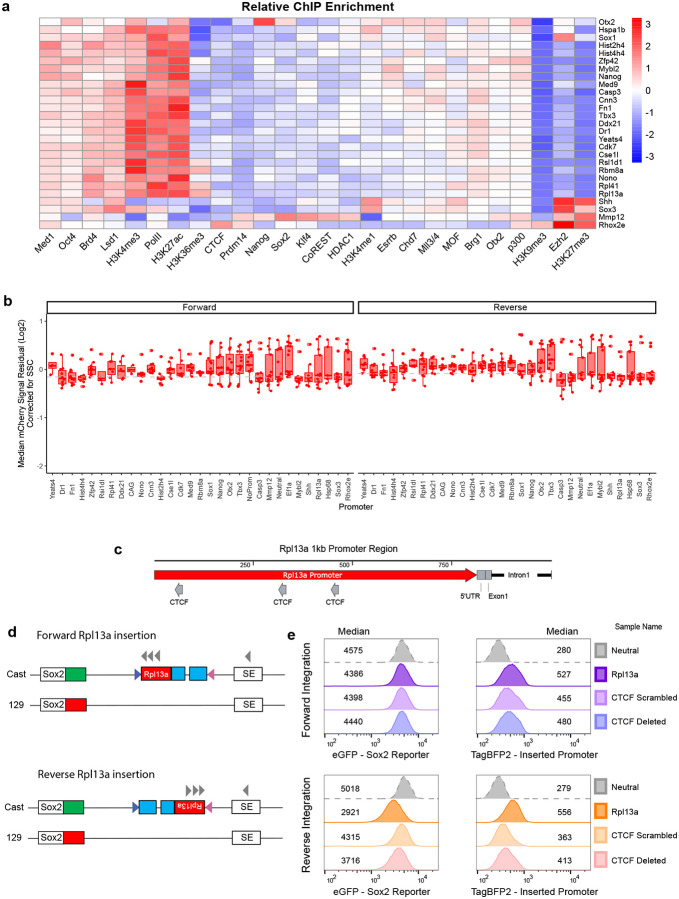
Additional information on selected promoters and screen outliers. **a)** Heatmap showing relative normalized ChIP-seq enrichment of histone modification, TF and COF binding profiles at 1 kb promoter regions used in the screen. Fill represents log coverage. **b)** Boxplot of median mCherry residual signal for each promoter after correction for SSC with points of each independent observation. Data includes 3 biological (from three independent clonal lines) and 3 technical replicates for each promoter. Boxes represent the median, IQR and whiskers (±1.5 IQR). **c)** Schematic of Rpl13a promoter region used in this study and CTCF motifs. Direction of the arrow shows orientation of the CTCF binding sites. **d)** Schematic of Rpl13a promoter integrated in the landing pad. Arrows show directionality of CTCF sites in Rpl13a and SE. **e)** Flow cytometry measurement histograms of eGFP and mTagBFP2 for Rpl13a mutant promoters integrated at the landing pad. Numbers represent median measurements.

**Figure 1 –:**
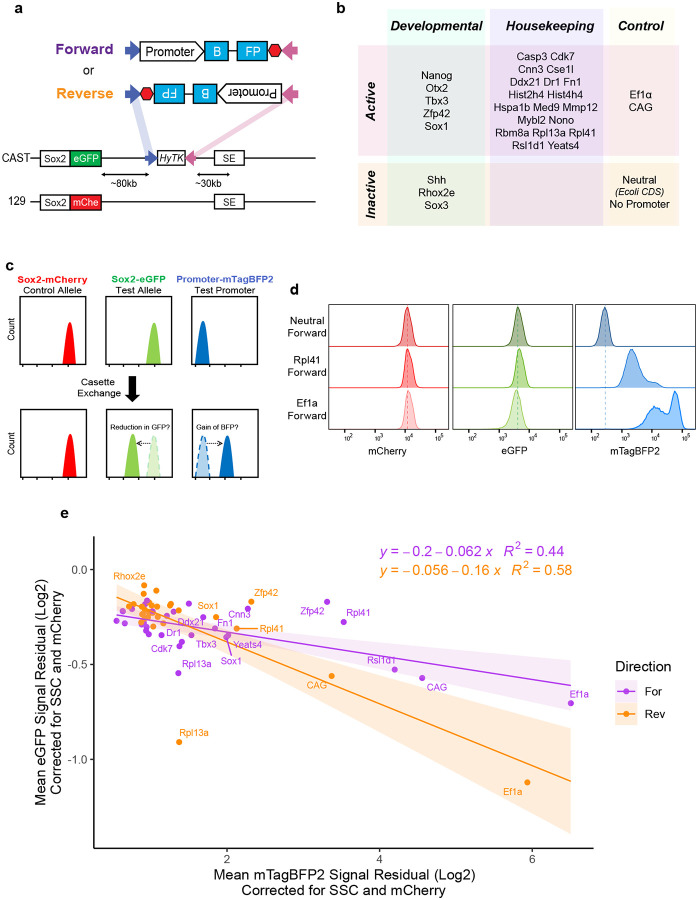
Promoter competition screen at the Sox2 locus. **a)** Schematic of Sox2 locus with the landing pad with hygromycin phosphotransferase-thymidine kinase fusion gene HyTK, flanked by heterospecific FRT sites, and of the inserts that are integrated at those sites either in the forward or in reverse orientation. Blue arrow represents FRT, pink arrow FRT3. SE, Super-enhancer. **b)** List of promoters tested. **c)** Mock flow cytometry histogram data demonstrating expected changes in fluorescent reporter signal in the competition scenario. **d)** Sample flow cytometry data from three integrations, representing neutral sequence and two tested promoters. Dashed lines are at the median of neutral insert. **e)** Scatterplot of mean corrected eGFP and mTagBFP2 signal from all promoters. Each dot is the average value from 3 biological replicates (measurements from 3 independent clonal lines), each measured in triplicate. The values are the mean of the residual for fluorescence intensity after correcting for SSC and mCherry (see methods). Equation and R^2^ value for linear regression for each direction is displayed.

To systematically introduce a new promoter at the *Sox2* locus, we used a previously generated Cast/129 hybrid mESC line, with the *Sox2* gene tagged with P2A-eGFP on the Cast allele and P2A-mCherry on the 129 allele^18^. To enable the controlled insertion of diverse promoters at a consistent location, we took advantage of the landing pad with heterospecific Flippase Recognition Sites (FRT and FRT3) in between *Sox2* and its SE on the eGFP/Cast allele (Fig.1a)^18^. To target our inserts to the landing pad, we generated donor plasmids with matching heterospecific FRT sites flanking a 1kb promoter fragment (spanning 850 base pairs upstream and 150 bp downstream from the most prominent TSS), followed by the mTagBFP2 coding sequence with an intron, and an SV40 polyA signal (Fig.1a, Supplementary Table 1). At this landing pad, we clonally integrated a small library, representing housekeeping and developmental promoters, the latter of which are either active or inactive in mESCs (Fig. 1b). In their endogenous context, selected active promoters were marked by H3K4me3, H3K27ac and RNA Polymerase 2 (Pol2), the inactive promoters were enriched for H3K27me3 or H3K9me3, and overall, the selected promoters had diverse profiles with respect to transcription factor and cofactor binding (Extended Data Fig. 1a). In addition, we integrated positive control sequences corresponding to the strong promoters Ef1a and CAG, and negative controls, with either no promoter insert or an E. coli coding sequence (CDS) that lacks regulatory activity in eukaryotic cells (hereafter referred to as the ‘neutral’ sequence) (Fig. 1b). All sequences were integrated in forward or reverse orientation (Fig. 1a), and multiple clonal mESCs lines were isolated for each insert integration. This synthetic platform allows us to simultaneously measure activity of the ectopically inserted promoter (using the mTagBFP2 signal as a proxy), the activity of the endogenous *Sox2* promoter located in cis from the insertion (using the eGFP signal as a proxy) and – as a reference – activity of the endogenous *Sox2* promoter at the allele without the landing pad (via the mCherry). In the promoter competition scenario, we expect the GFP signal to decrease in the presence of the BFP signal, whereas the mCherry should be unaffected (Fig. 1c).

### Promoter competition is dependent on the strength and orientation of the inserted promoter

To explore promoter competition, we assayed three clonal lines from each promoter insert in both forward and reverse directions and measured mCherry, eGFP and mTagBFP2 fluorescence intensity using flow cytometry. As expected, mCherry signal was stable across the cell lines with different promoter integrations (Extended Data Fig. 1b). We next plotted the corrected mean fluorescence intensity (See Methods) for eGFP and mTagBFP2 (Fig. 1e) and observed an inverse correlation. That is, with increasing mTagBFP2 levels from the inserted promoter, the expression driven by the endogenous *Sox2* promoter was progressively reduced, suggesting that promoter strength (as approximated here by the mTagBFP2 expression) is one of the determinants of competition (Fig. 1d,e). Interestingly, the extent of the competition was also dependent on the orientation of the promoter insert, as demonstrated by the differences in the slope of two linear regression fits for forward and reverse integrations (Fig. 1e). Specifically, promoters in the reverse orientation (facing the *Sox2* promoter) showed more competition than their counterparts in the forward orientation (i.e. same orientation as the *Sox2* promoter). Notably, we also observed stronger mTagBFP2 expression in the forward compared to the reverse orientation for the respective promoter insert pairs, and we will come back to this observation when discussing Figure 7. Altogether, these results suggest that promoter competition is dependent both on the strength and the orientation of the inserted promoter.

### Promoter competition can be modulated by the presence of CTCF sites in the promoter

We were intrigued by the one strong outlier to the trendline, which was the Rpl13a promoter in the reverse orientation, that had noticeably reduced *Sox2* eGFP despite a moderate level of mTagBFP2 expression (Fig. 1e). To explore possible reasons for this effect, we investigated genomic features of the Rpl13a promoter. We noted the presence of three tandem CTCF binding motifs oriented in the same direction (Extended Data Fig. 1c). We posited that these CTCF sites may disrupt communication of the SE with the endogenous *Sox2* promoter by creating a new boundary and blocking cohesin-mediated loop extrusion (Extended Data Fig. 1d). Indeed, previous work showed that tandem CTCF sites in a convergent orientation to those at the SE can attenuate *Sox2* expression^18^; such convergent orientation is present in the reverse direction insert (Extended Data Fig. 1d). In contrast, in the forward direction, the CTCF binding sites in the Rpl13a promoter would be in a divergent orientation, rendering CTCF unable to stop cohesin extruding from the SE (Extended Data Fig. 1d). To test if CTCF sites contribute to the Rpl13a outlier effect, we integrated mutant versions of the Rpl13a promoter, in which we either deleted or scrambled the CTCF motifs. In forward Rpl13a integration, the mutant CTCFs had a negligible effect on both mTagBFP2 and eGFP signal relative to the unmodified Rpl13a. Yet in reverse direction, deletion or scramble of CTCF motif both led to decrease of mTagBFP2 and rescued the eGFP reduction (Extended Data Fig. 1e). This implies that Rpl13a in reverse orientation can stably loop with SE and induce more mTagBFP2, while also disrupting the Sox2-SE relationship and lowering eGFP levels. These data argue that presence and orientation of CTCF sites at the promoters can influence promoter competition.

### Allelic series of Ef1a promoter augments correlation between promoter strength and competition

**Extended Data Fig. 2 –:**
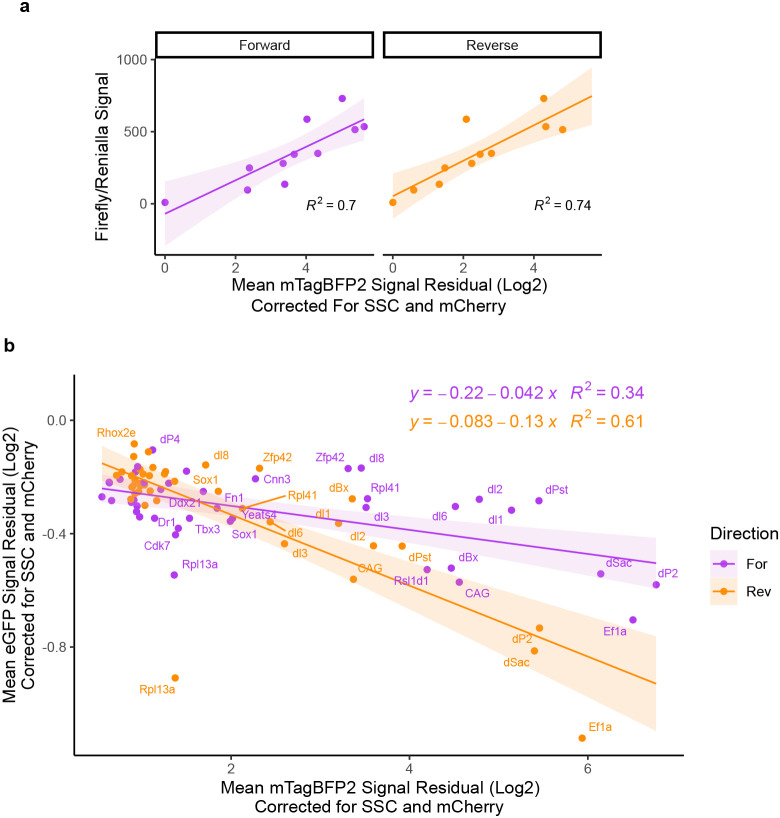
Promoter competition is driven by the strength of the competing promoter. **a)** Correlation plot of promoter strength measured in the episomal luciferase reporter assay compared to strength of the integrated promoter measured by mTagBFP2 output. Coefficient of determination R^2^ values for linear regression are displayed. **b)** Scatterplot of corrected mean eGFP and mTagBFP2 signal of all promoters tested, for 3 biological replicates (independent clonal lines), measured in triplicate. Equation and R^2^ value for linear regression for each direction is displayed.

**Figure 2 –:**
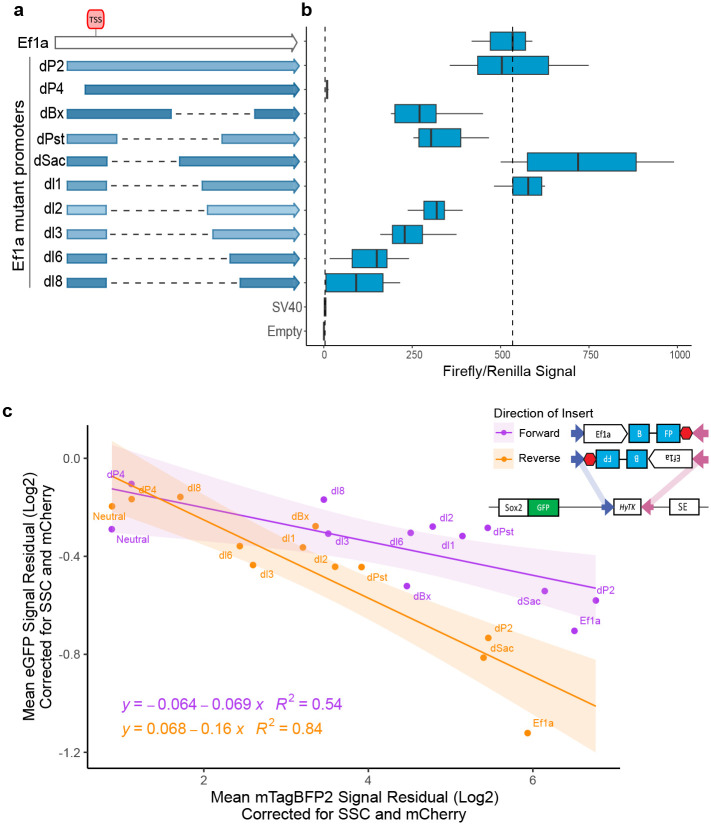
Allelic series of Ef1a promoter recapitulates inverse correlation between expression from integrated and endogenous promoters. **a)** Schematic of allelic series of Ef1a promoter, dashed lines signify deleted regions. **b)** Episomal Luciferase reporter assay measurements from each mutant promoter. Measurements performed twice with two distinct preps of reporter plasmid, transfected in duplicate. Firefly divided by Renilla signal is displayed. Boxes represent the median, IQR, and whiskers (±1.5 IQR). **c)** Scatterplot of mean corrected eGFP and mTagBFP2 signal of mutant Ef1a promoters for 3 biological replicates (independent clonal cell lines), measured in triplicate. Equation and R^2^ value for linear regression for each direction is displayed. Schematics of integration in forward and reverse orientation is shown in the inset.

To confirm that promoter strength is the main driver of competition, we selected our strongest performing promoter, Ef1a, and synthesized deletion mutants that modulated its strength (Fig. 2a)^19^. First, we confirmed that these mutant promoters have varying levels of activity in an episomal luciferase reporter assay, with over an order of magnitude difference between the strongest and the weakest version of the E1fa promoter (Fig. 2b). Next, we inserted these promoters clonally into our landing pad, and measured mTagBFP2, eGFP and mCherry levels. As expected, the mTagBFP2 signals were well correlated with the activity of the respective mutant promoters in luciferase assay (Extended Data Fig. 2a). Importantly, we once again observed the inverse correlation between eGFP and mTagBFP2 levels, suggesting that the competition phenotype is driven by the strength of the promoter (Fig. 2c). We next plotted all promoters from Figures 1 and 2 together, which shows the relationship between promoter strength and competition over a broad range of promoter types and activities (Extended Data Fig. 2b).

### Loss of HUSH-mediated silencing enhances promoter competition

**Figure 3 –:**
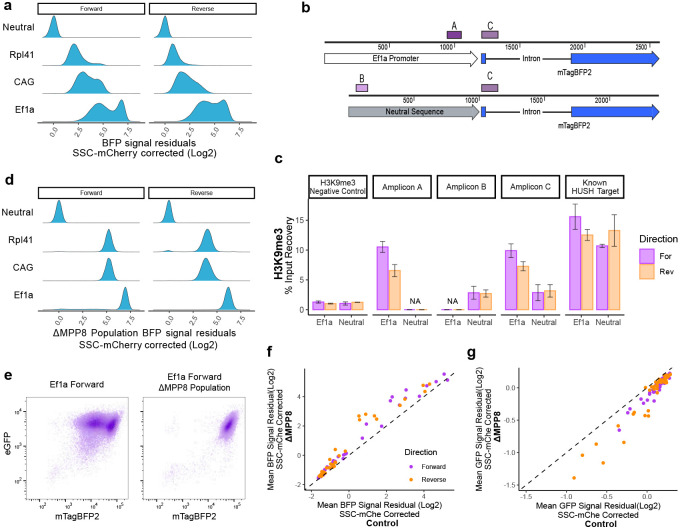
HUSH mediated silencing at promoter inserts. **a)** Density plot of mTagBFP2 residual after correction for SSC and mCherry for a subset of promoters integrated in the WT background. **b)** Schematic of inserts with location of ChIP-qPCR amplicons indicated with purple bars. **c)** H3K9me3 ChIP-qPCR percent input recovery at amplicon sites labeled in (B). n=4 for Ef1a, n=2 for Neutral inserts, mean ± s.e. **d)** Density plot of mTagBFP2 residual after correction for SSC and mCherry for a subset of promoters in ΔMPP8 populations. **e)** Raw flow measurements of eGFP and mTagBFP2 signal in Ef1a Forward insert in WT and ΔMPP8 backgrounds. **f)** Scatterplot comparing mean corrected mTagBFP2 signal in ΔMPP8 and control edited populations. Dashed line, y=x. **g)** Scatterplot comparing mean corrected eGFP signal in ΔMPP8 and control edited populations. Dashed line, y=x.

Upon closer inspection of the mTagBFP2 signal from the inserted promoters, we observed that many promoters displayed a bimodal expression pattern, with high- and low-expressing populations present (Fig. 3a), reminiscent of variegated transgene silencing by the Human Silencing Hub Complex (HUSH)^16,20^. HUSH is a transcription-dependent silencing complex that targets non-self DNA such as transgenes or transposable elements^20^. Once HUSH recognizes foreign transcripts, it mediates deposition of H3K9me3 to attenuate their expression^20^. This was surprising, given that we have included an intron in the mTagBFP2 coding sequence, a feature which was shown to counteract HUSH silencing^21^. To investigate if the bimodal mTagBFP2 expression may be related to HUSH-driven H3K9me3 deposition, we performed ChIP-qPCR to measure H3K9me3 enrichment at the reporter sequences using mESC lines with either the Ef1a promoter or the neutral (non-expressing) sequence inserted in the forward or reverse orientations (Fig. 3b). In each of the four cell lines we tested five amplicons, including H3K9me3-negative and positive regions outside of the *Sox2* locus, an amplicon unique to each of the inserted promoter sequences (either Ef1a Amplicon A or neutral sequence Amplicon B), and an amplicon corresponding to the exon-intron junction of the inserted mTagBFP2 reporter (shared between all four cell lines, Amplicon C) (Fig. 3b). We detected above-background enrichment of H3K9me3 at both Ef1a and neutral sequence reporters irrespective of orientation, although H3K9me3 deposition was substantially higher at the Ef1a reporter compared to a neutral sequence (Fig. 3c).

**Extended Data Fig. 3 –:**
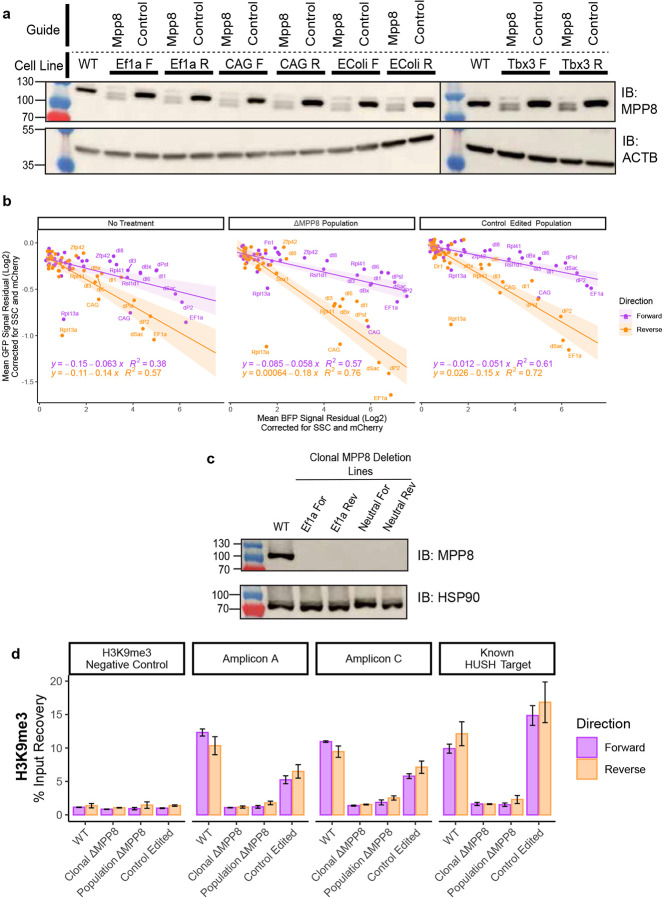
Controls for HUSH ablation in landing pad cells. **a)** MPP8 western blot in WT, and a subset of MPP8 targeted, and Control targeted populations. Membranes were probed with antibodies against MPP8 and ACTB as a loading control. **b)** Scatterplot of corrected eGFP and mTagBFP2 signal for all promoters tested in WT, ΔMPP8 and control edited backgrounds. ΔMPP8 and control edited measurements were done in triplicates in population of cells. **c)** MPP8 western blot in WT, and clonal ΔMPP8 lines. Membranes were probed with antibodies against MPP8 and HSP90 as a loading control. **d)** H3K9me3 ChIP-qPCR percent input recovery at sites labeled in (3B) for Ef1a insert lines in clonal WT, clonal ΔMPP8, population ΔMPP8 and control edited populations. n=2, mean ± s.e.

We hypothesized that increased deposition of H3K9me3 at the active Ef1a reporter may be related to the fact that establishment of HUSH silencing requires transcription^17,22^, and thus that the observed bimodal expression of the mTagBFP2 reporters may be a consequence of HUSH activity. To test this, we knocked out MPP8, a core HUSH subunit, in the Ef1a promoter insert lines (Extended Data Fig. 3a). We indeed observed loss of the low mTagBFP2 population (Fig. 3d, compare to 3a, and Fig. 3e) and loss of H3K9me3 at the reporter (Extended Data Fig. 3d). Similarly, knockout of MPP8 led to loss of bimodal expression from reporters driven by other promoters, such as CAG and Rpl41 (Fig. 3d). Thus, surprisingly, even in the presence of an intron and a strong promoter, the inserted reporters are susceptible to HUSH-mediated silencing that may confound our understanding of promoter competition.

We next examined how loss of HUSH affects promoter competition. To this end, we knocked out MPP8 in our promoter insert lines by stable expression of Mpp8-targeting guides and Cas9. We confirmed that this strategy results in successful depletion of MPP8 protein at the population level by performing immunoblot analysis on a subset of promoter integration lines (Extended Data 3a). We then measured mTagBFP2, eGFP, and mCherry levels in these populations and saw that removal of HUSH did not change the inverse correlation between eGFP and mTagBFP2 (Extended Data 3b). As expected – given the results above – loss of MPP8 yielded an increase in the mean mTagBFP2 expression from all promoters compared to the nontargeting guide-treated control cells (Fig. 3f). Conversely, expression of eGFP driven by the *Sox2* promoter was decreased across all ΔMpp8 cells compared to controls (Fig. 3g). Taken together, in the absence of HUSH-mediated silencing, we observe elevated expression from the inserted promoters and increased competition with the *Sox2* promoter.

### Promoter competition is position dependent

**Figure 4 –:**
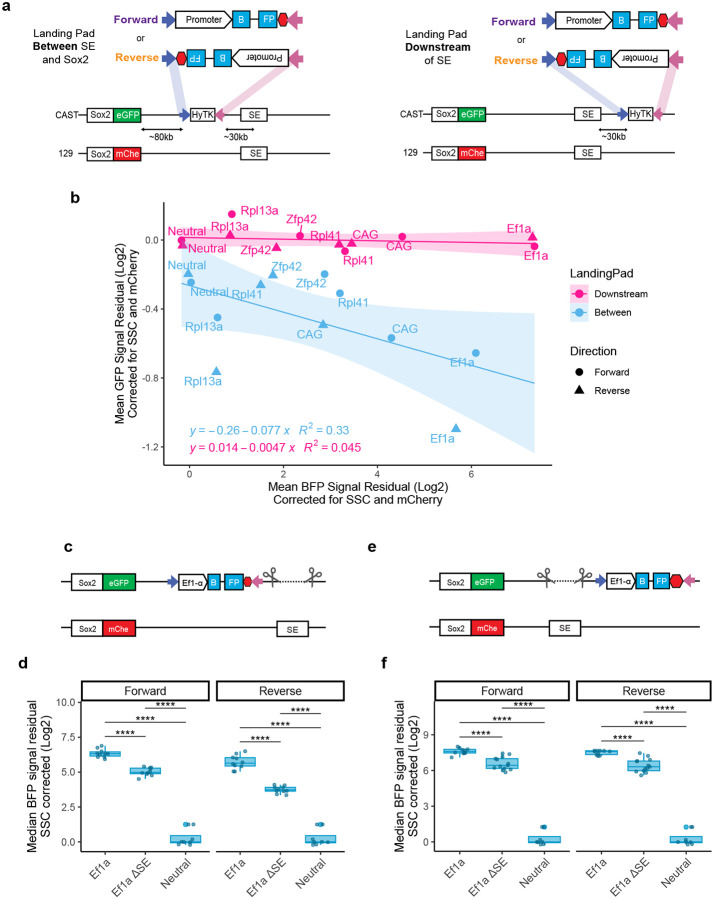
Promoter competition is position dependent. **a)** Schematic comparing two landing pad locations: between SE and Sox2 gene (left) and downstream of the SE (right). **b)** Scatterplot of corrected mean eGFP and mTagBFP2 signal for subset of promoters tested in both at the “between” and “downstream” landing pads. Mean of 3 biological replicates, measured in triplicate. Values shifted vertically and horizontally to make neutral insert adjusted to 0. **c)** Schematic of allele-specific SE deletions in cell lines with the Ef1a promoter integration at the “between” landing pad. **d)** Corrected median mTagBFP2 measurements from in between landing pad lines with Ef1a integrated, Ef1a integrated with ΔSE and neutral insert controls. Median from 3 biological replicates (independent clonal lines), measured in triplicates plotted together. Boxes represent the median, IQR, and whiskers (±1.5 IQR). Pairwise t-tests with Bonferroni correction, ****Padj ≤ 0.0001. **e)** Schematic of allele specific SE deletions in cell lines with the Ef1a promoter integration at the “downstream” landing pad. **f)** Corrected median mTagBFP2 measurements from downstream landing pad lines with Ef1a integrated, Ef1a integrated with ΔSE and neutral insert controls. Median from 3 biological replicates (independent clonal lines), measured in triplicates plotted together. Boxes represent the median, IQR, and whiskers (±1.5 IQR). Pairwise t-tests with Bonferroni correction, ****Padj ≤ 0.0001. In **d-f** values shifted vertically to make neutral insert adjusted to 0.

Next, we asked whether the competition is dependent on the location of the competing promoter between the endogenous promoter and the SE. We used an alternative version of the parental cell line, which has the landing pad at an equidistant location (~30kb away) but downstream from the SE, instead of in between the SE and *Sox2* (Fig. 4a)^18^. We integrated a subset of the promoters at the downstream landing pad and assayed their activity alongside their in-between landing pad counterparts. As expected, promoters inserted at the downstream landing pad showed varied levels of activity, as measured by the mTagBFP2 (Fig. 4b). However, in contrast to their in-between counterparts, they had no effect on the expression from the *Sox2* promoter, as measured by eGFP (Fig. 4b), indicating that promoter competition is indeed dependent on the genomic location. Of note, the integrations at the downstream landing pad often showed higher mTagBFP2 expression than their in-between counterparts, which may suggest a reciprocal competition between *Sox2* and the integrated promoter.

**Extended Data Fig. 4 –:**
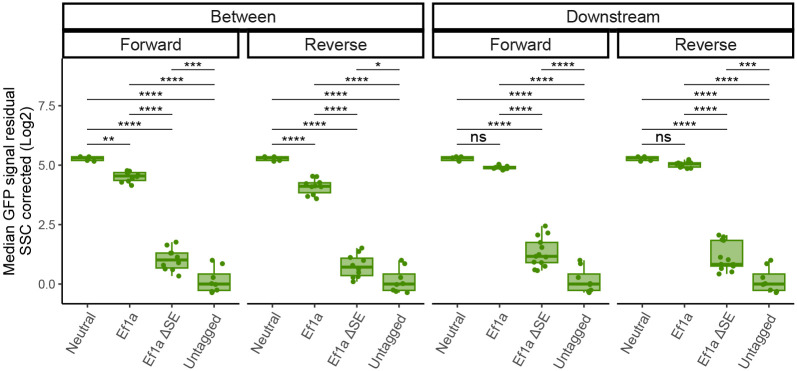
Changes in eGFP levels in response to SE deletion. Corrected median eGFP measurements from in between and downstream landing pad lines with neutral insert integrated, Ef1a integrated, Ef1a integrated with ΔSE and untagged WT control. Median from 3 biological replicates (independent clonal lines), measured in triplicates plotted together. Values shifted vertically to make untagged control adjusted to 0. Boxes represent the median, IQR, and whiskers (±1.5 IQR). Pairwise t-tests with Bonferroni correction, *Padj ≤ 0.05; **Padj ≤ 0.01; ***Padj ≤ 0.001; ****Padj ≤ 0.0001.

Although the downstream landing pad is within the same TAD as *Sox2* and SE^23^, it lies outside of the previously reported Sox2-SE loop contact demarcated by the convergent CTCF sites^24,25^. We therefore considered a possibility that the promoter integrated at the downstream site does not receive regulatory input from the SE and hence, does not compete for the limited resources of SE with the endogenous *Sox2* promoter. To test this, we deleted SE at the Cast allele (that is, the allele containing the landing pad and *Sox2-eGFP* reporter) in cell lines in which the Ef1a promoter was integrated at either the in-between or at the downstream landing pad (Fig. 4c,e). As expected, given the dominant role of the SE in regulating *Sox2* expression in mESCs, most of the eGFP expression was lost upon SE deletion across all tested cell lines (Extended Data Fig. 4a). ‘Interestingly, while the median mTagBFP2 expression was overall higher at the downstream landing pad location, the deletion of the SE impacted the mTagBFP2 levels regardless of the location (Fig. 4d, f). These data indicate that despite its strong inherent activity, expression from the Ef1a promoter is comparably boosted by the SE in both locations. With a simple enhancer resource sharing model of competition, it would be expected that any inserted promoter boosted by the SE must be sequestering the limited resources that the cognate promoter relies on and should therefore display promoter competition. Under such model, both locations of Ef1a promoter should result in competition. Given that only the in-between location results in competition, our results argue against a simple enhancer resource sharing model underlying promoter competition.

### Cohesin accumulates at the active reporter but is not required for competition

**Extended Data Fig. 5 –:**
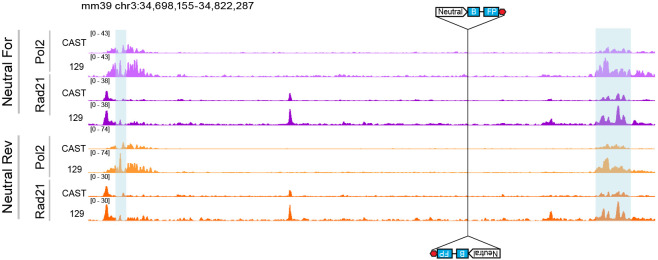
Cohesin does not accumulate near neutral sequence insert. Allele specific RNA Pol2 and Rad21 ChIP-seq tracks in the Neutral sequence integration cell lines. Landing pad location and direction marked with cartoon of Neutral control integration, blue highlights mark Sox2 coding sequence and SE, respectively.

**Figure 5:**
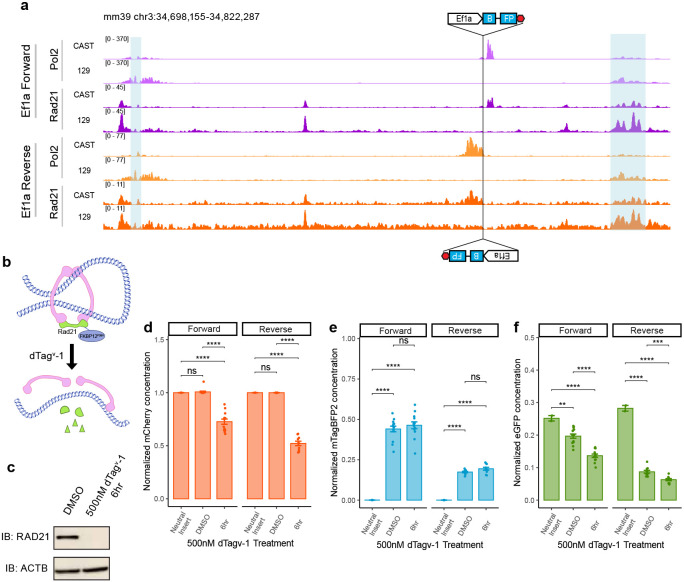
Loss of cohesin does not eliminate competition. **a)** Allele specific RNA Pol2 and Rad21 ChIP-seq tracks in Ef1a integration cell lines. Landing pad location and direction marked with cartoon of Ef1a integration, blue highlights mark Sox2 coding sequence and SE. **b)** Schematic of RAD21 depletion upon dTag^v^-1 addition. **c)** RAD21 western blot in representative Rad21-FKBP12^F36V^ clonal line in DMSO control and after 6 hr depletion. Membranes were probed with antibodies against RAD21 and b-actin as a loading control. **d)** RT-qPCR measurements of mCherry transcripts after 6 hrs of RAD21 depletion. **e)** RT-qPCR measurements of mTagBFP2 transcripts after 6 hrs of RAD21 depletion. **f)** RT-qPCR measurements of eGFP transcripts after 6 hrs of RAD21 depletion. In **d-f** Absolute concentrations were first normalized to housekeeping gene Gapdh, then normalized to the corresponding mCherry control. 6 clonal cell lines were assayed for each direction, in duplicate, mean ± s.e. Pairwise t-tests with Bonferroni correction, *Padj ≤ 0.05; **Padj ≤ 0.01; ***Padj ≤ 0.001; ****Padj ≤ 0.0001.

Given the privileged role of the in-between location for promoter competition, we explored the possibility that transcription of the integrated promoter and/or its associated transcriptional machinery may be acting as an obstacle to cohesin mediated loop-extrusion, hindering the communication of the *Sox2* promoter and the SE^26^. Our results with the Rpl13a promoter show that increased competition can result from the presence of tandem CTCF sites ^18^ and CTCF binding is a known barrier to loop extrusion ^27-29^. While the Ef1a promoter lacks CTCF binding, as do most promoters examined in our study, it has been suggested that RNA Polymerase II (Pol2) may also act as a barrier to cohesin mediated loop extrusion ^30-34^. To investigate changes in the localization of Pol2 and Rad21, a cohesin subunit, in the presence of the reporter integration, we performed ChIP-seq against Pol2 and Rad21 in cell lines with Ef1a or the inactive, neutral sequence reporter. To avoid confounding effects from HUSH-mediated silencing, we used cell lines in which MPP8 has been knocked out clonally (see Extended Data Fig. 3c and 3d for validation). We leveraged SNPs between Cast and 129 strains to map reads in an allele specific manner. In our Forward Ef1a integrated cell lines we detected a pile-up of Pol2 at the 3’ end of the integration, where Pol2 is likely terminating (Fig. 5a), uniquely at the Cast allele. The pileup was on the opposite side in the Reverse Ef1a cell lines, once again demarcating the 3’ region downstream from the integration. Interestingly, we also observed Rad21 peaks that co-occur with Pol2 (Fig. 5a) near the integration. These peaks were not present on the 129 allele, nor at the Cast allele in cells where the neutral sequence reporter has been integrated, indicating that the presence of an active promoter is required for cohesin accumulation at this site (Extended Data Fig. 5a).

To test how cohesin depletion affects promoter competition, we tagged Rad21 with an FKBP12^F36V^ degron tag in the Ef1a reporter cell lines, permitting a rapid depletion of cohesin upon addition of the degron-inducing dTag^v^-1 molecule^35^ (Fig. 5b,c). Since Rad21 is essential for cell cycle progression, we could not culture cells for more than one cell division following dTag^v^-1 addition, which would be necessary to assay the effects on the fluorophore reporters at the protein level. Instead, we depleted Rad21 for 6 hours and measured *mCherry, eGFP* and *mTagBFP2* transcript levels using RT-qPCR (Fig. 5d-f). Loss of cohesin resulted in a decrease in *mCherry* levels, consistent with the previous reports indicating that cohesin loss decreases *Sox2* transcription by ~25%^36^ (Fig. 5d), and it led to a modest (but statistically insignificant) increase in *mTagBFP2* expression (Fig. 5e). If loss of cohesin would alleviate promoter competition, then *eGFP* transcript levels should recover in dTag^v^-1-treated samples compared to the DMSO treated controls, even after accounting for the decreases associated with the effect of cohesin on the endogenous *Sox2* expression, as measured by the reduction in *mCherry.* However, the effects of cohesin depletion are comparable at the *mCherry* and the *eGFP* transcripts, with a reduction of *eGFP* expression in both orientations (Fig. 5f). This argues that cohesin is not necessary for competition.

### Blocking transcription downstream from the TSS rescues promoter competition

**Figure 6 –:**
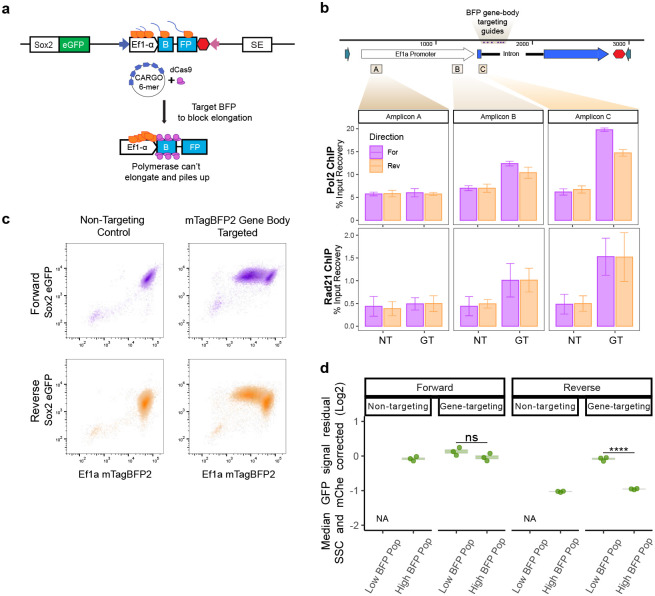
Blocking transcription with dCas9-CARGO rescues competition. **a)** Schematic of dCas9-CARGO strategy. A 6-mer CARGO guide RNA array targeting the gene body of mTagBFP2 and dCas9 is introduced to the cells with the goal of blocking elongation. **b)** RNA Pol2 and Rad21 ChIP-qPCR percent input recovery at highlighted amplicon sites. n=3, mean ± s.e is plotted, NT=Non-targeting control, GT=Gene targeting CARGO. CARGO guides’ target locations are labeled with purple lines and highlighted in beige. **c)** Flow measurements of eGFP and mTagBFP2 signal in Ef1a promoter insert cells expressing dCas9 and either non-targeting control or gene-targeting CARGO array from a representative replicate. **d)** Corrected median eGFP measurements from “Low BFP” and “High BFP” gates in non-targeting and gene-targeting populations. Median of measurements from 3 replicates plotted. Boxes represent the median, IQR, and whiskers (±1.5 IQR). Pairwise t-tests with Bonferroni correction, ****Padj ≤ 0.0001.

Since the loss of cohesin does not alleviate competition, we wondered if blocking transcription through the inserted reporter would, as our data shows correlation between competition and promoter strength (and in effect, transcript levels). To test if blocking transcription can rescue the effect of competition and recover endogenous *Sox2* eGFP levels, we employed our previously described CARGO approach.^37^ We assembled an array of six co-directional guides targeted to the 5’ intron region of the *mTagBFP2* sequence (Fig. 6a) that will recruit dCas9, with a goal of physically blocking passage of Pol2 through the gene without affecting assembly of transcriptional machinery at the promoter^38^. We first introduced this gene targeting (GT) CARGO array – or a control, non-targeting (NT) CARGO array – using PhiC integrase, followed by insertion of a catalytically inactive Cas9 lacking any effector domain (dCas9) via a PiggyBac vector. Once again, we used the Ef1a reporter or neutral sequence parental cell lines in which MPP8 has been knocked out (Extended Data Fig. 3c) to avoid confounding effects of HUSH silencing.

**Extended Data Fig. 6 –:**
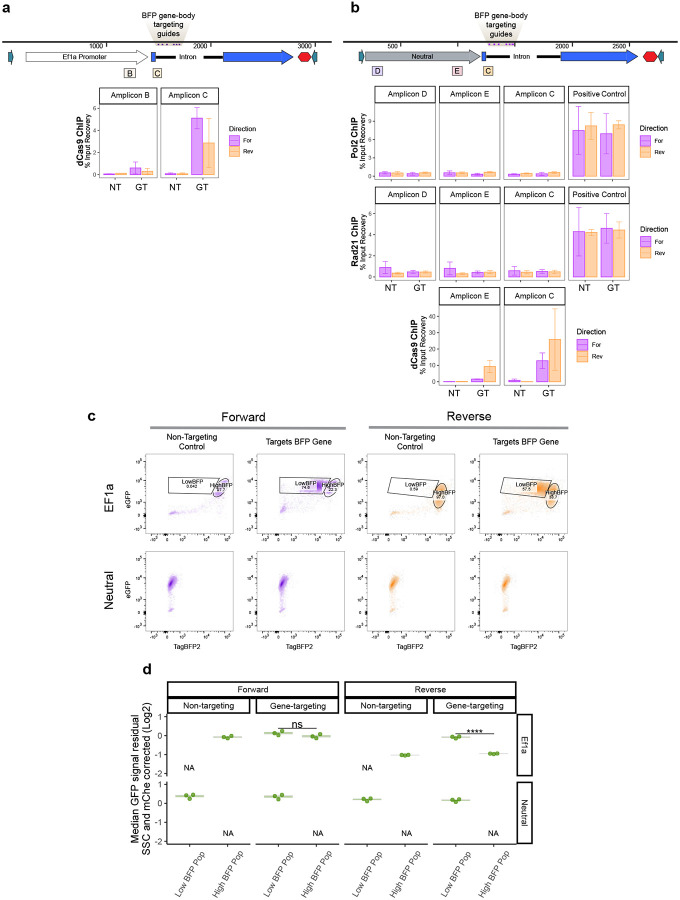
dCas9 CARGO controls in neutral insert line. **a)** dCas9 ChIP-qPCR percent input recovery at highlighted amplicon sites. n=2, mean ± s.e is plotted, NT=Non-targeting control, GT=Gene targeting CARGO. **b)** RNA Pol2, Rad21 and dCas9 ChIP-qPCR percent input recovery at highlighted amplicon sites in neutral integration lines. n=2 for dCas9, n=3 for Pol2 and Rad21, mean ± s.e is plotted.**c)** Flow cytometry data showing “Low BFP” and “High BFP” gates in NT and GT populations for both Ef1a and neutral insert lines from representative replicate. All data from neutral integrations are considered “Low BFP”. **d)** Corrected eGFP measurements from “Low BFP” and “High BFP” gates in non-targeting and gene-targeting populations, for both Ef1a and neutral insert lines. Corrected median eGFP measurements from “Low BFP” and “High BFP” gates in non-targeting and gene-targeting populations. Median of measurements from 3 replicates plotted. Boxes represent the median, IQR, and whiskers (±1.5 IQR). Pairwise t-tests with Bonferroni correction, ****Padj ≤ 0.0001.

We first performed dCas9 ChIP-qPCR to confirm that dCas9 accumulates at the targeted site in the GT CARGO, but not in the NT CARGO expressing cells (Extended Data Fig. 6a). Our assay was designed to create an obstacle for elongating Pol2 downstream from the initiation site, and thus in our GT CARGO expressing cells, Pol2 should accumulate downstream from the TSS and upstream from the targeted site. Indeed, Pol2 ChIP-qPCR analysis showed increased Pol2 levels upstream of the targeted site (Amplicons B and C), but not at the TSS (Amplicon A) in the GT-CARGO compared to NT CARGO expressing cells (Fig. 6b). Interestingly, cohesin also accumulated at this site along with Pol2, as revealed by the Rad21 ChIP-qPCR (Fig. 6b). In contrast, in our neutral sequence control cell lines, we observed enrichment of dCas9 but not of Pol2 or Rad21 signal in GT CARGO cells, indicating that dCas9 binding in the absence of transcription does not stall cohesin (Extended Data Fig. 6b).

Next, we tested the consequences of these perturbations on gene expression. Consistent with the transcriptional block, in a large subpopulation of cells transfected with the GT CARGO, but not with the NT CARGO, we observed a strong reduction of mTagBFP2 signal (“LowBFP”) (Fig. 6c, Extended Data Fig. 6c). Notably “HighBFP” subpopulation was also present, likely corresponding to cells in which the locus was not effectively targeted (Fig. 6c, Extended Data Fig. 6c). We took advantage of this heterogeneity and gated the two subpopulations to analyze their eGFP signals. We observed that LowBFP population had higher eGFP signal than HighBFP population, and this effect was more striking in the Reverse Ef1a promoter orientation, which is associated with higher competition (Fig. 6d, Extended Data Fig. 6d). These results indicate that blocking transcription through the reporter downstream from the transcription initiation site alleviates promoter competition and rescues endogenous *Sox2* eGFP levels. This is true even in the increased presence of Pol2 and cohesin at the integration site, further demonstrating that cohesin accumulation is not sufficient to drive competition.

### Transcriptional read-through increases promoter competition

**Figure 7 –:**
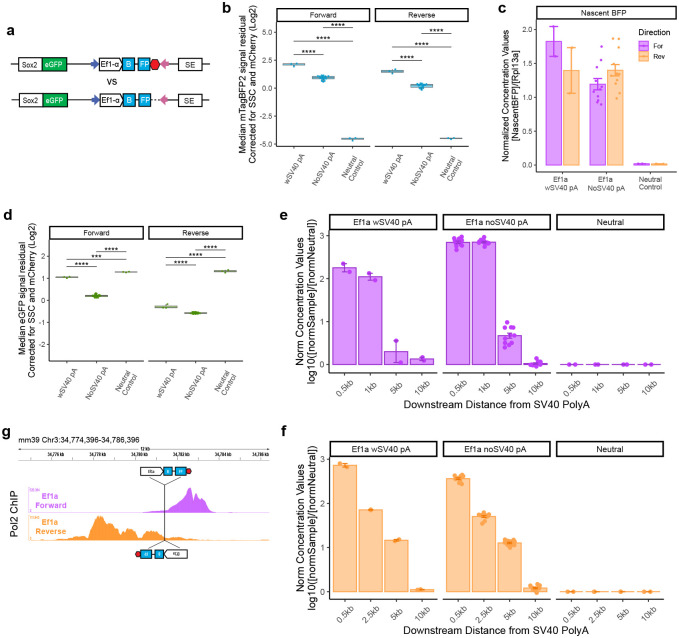
Transcript length affects competition. **a)** Schematic of Ef1a reporter integration with or without SV40 polyA signal (indicated in red). **b)** Corrected median mTagBFP2 signal from cells with Ef1a integrated with or without SV40 polyA. Two clonal lines with polyA were compared to 6 clonal lines without SV40 polyA. Flow measurements were repeated 3 times. Boxes represent the median, IQR, and whiskers (±1.5 IQR). Pairwise t-tests with Bonferroni correction, ****Padj ≤ 0.0001. **c)** RT-PCR analysis of nascent BFP transcript levels. Absolute concentration of Nascent BFP transcripts was normalized to housekeeping Rpl13a; 6 clones without SV40 polyA compared to 1 clone with Ef1a polyA and neutral polyA, in duplicate. mean ± s.e is plotted. **d)** Corrected median eGFP signal from cells with Ef1a reporter integrated with or without SV40 polyA. Two clones with polyA compared to 6 clones without SV40 polyA. Flow repeated 3 times. Boxes represent the median, IQR, and whiskers (±1.5 IQR). Pairwise t-tests with Bonferroni correction, ***Padj ≤ 0.001; ****Padj ≤ 0.0001. **e)** RT-PCR analysis of read-through transcript levels in forward Ef1a inserts. Absolute concentration of downstream transcripts was normalized to housekeeping Rpl13a, then normalized to concentration from neutral insert. 6 clones without SV40 polyA compared to 1 clone with Ef1a polyA and neutral polyA, in duplicate. mean ± s.e is plotted in log scale. **f)** RT-PCR analysis of read-through transcript levels in reverse Ef1a inserts. Absolute concentration of downstream transcripts was normalized to housekeeping Rpl13a, then normalized to concentration from neutral insert. 6 clones without SV40 polyA compared to 1 clone with Ef1a polyA and neutral polyA, in duplicate. mean ± s.e is plotted in log scale. **g)** Total (non-allele specific) RNA Pol2 ChIP-seq tracks zoomed near the landing pad in cells with the forward and reverse Ef1a integrations.

The observations that: (i) blocking transcription downstream from the TSS of the inserted promoter rescues competition, and (ii) competition is dependent on the strength (and thus RNA output) of the promoter, suggested to us that the transcript itself may be driving the competition. We wondered if in addition to transcript levels, other properties such as length may thus contribute to the observed effects. To promote transcriptional read-through, we removed the SV40 polyA signal that promotes termination from our Ef1a reporter (Ef1a No-SV40pA) (Fig. 7a). We then reintegrated the reporter at the landing pad in both orientations, isolated clonal mESC lines, and assayed fluorophore expression in parallel with Ef1a still containing SV40 polyA (Ef1a wSV40 PolyA) and neutral-sequence reporter cell lines containing an intact SV40 polyA (Fig. 7a).

**Extended Data Fig. 7 –:**
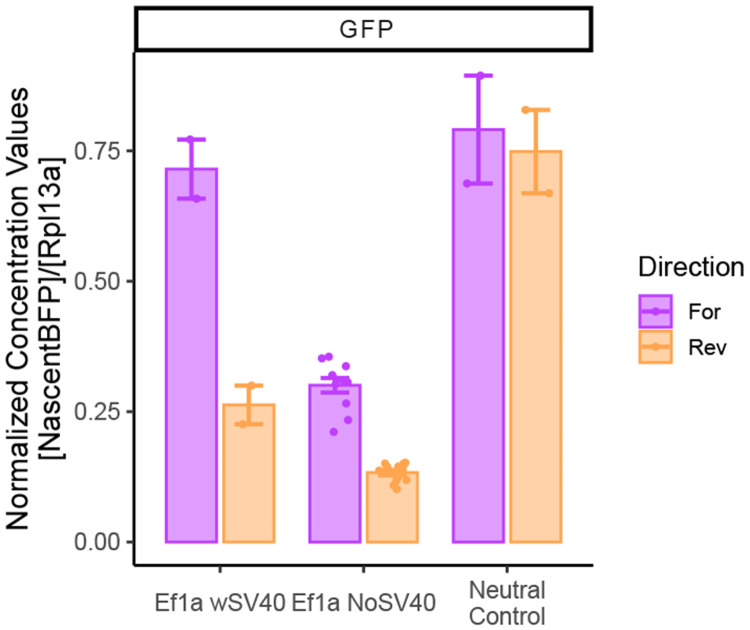
The effects of SV40 polyA on eGFP transcript levels. RT-PCR analysis of eGFP transcript levels. Absolute concentration of eGFP transcripts normalized to housekeeping Rpl13a. 6 clones without SV40 polyA compared to 1 clone with Ef1a polyA and neutral polyA, in duplicate. Mean ± s.e is plotted.

We first analyzed expression of mTagBFP2 by flow cytometry and observed decreased mTagBFP2 levels in the No-SV40pA reporter cells (Fig. 7b). However, this measurement may be confounded by the fact that loss of polyadenylation signal may affect mRNA stability or translation^39^. We therefore assayed the nascent *mTagBFP2* RNA levels by RT-qPCR, leveraging the intron in the *mTagBFP2* sequence. These measurements showed no effect of polyA deletion on nascent *mTagBFP2* RNA level when the reporter was integrated in the reverse orientation, and a modest decrease when the reporter was integrated in the forward orientation (Fig. 7c). Next, we analyzed eGFP expression by flow cytometry and observed that loss of the polyA sequence from the reporter was associated with further decrease in eGFP level (Fig. 7d). Concordant results were obtained when the *eGFP* mRNA was analyzed by RT-qPCR (Extended Data Fig. 7). These observations are consistent with the notion that increased transcript length may lead to increased competition.

We noted that the removal of the polyA signal caused a greater reduction of the eGFP signal in the forward direction (Fig. 7d, Extended Data Fig. 7). We wondered whether the difference in the transcriptional read-through length after the SV40 polyA signal could explain this observation. We designed primers tiling up to 10kb following the SV40 polyA signal, and profiled RNA expression by RT-qPCR, comparing the neutral sequence reporter (which should have no detectable transcription and serves as a reference) and the Ef1a reporter with or without polyA, in both orientations. In the forward orientation, removal of the polyA signal resulted in elevated transcript levels up to 5 kb downstream from where the polyA would be (Fig. 7e). Importantly, we did not detect much transcription 10 kb downstream from the polyA, suggesting that transcriptional interference due to a head-on collision with the Pol2 transcribing from the SE located 30 kb away which might disrupt SE activity is an unlikely explanation for the observed increase in competition (Fig. 7e).

Surprisingly, in the reverse orientation, we detected transcription up to 5 kb downstream from the polyA even in the polyA-containing reporter, with no further increases upon the polyA signal deletion (Fig. 7f). This is consistent with the smaller impact of polyA deletion in the reverse orientation, but also suggests that in the presence of the polyA signal, the reporter integrated in the reverse direction produces a longer transcript compared to the reporter integrated in the forward direction. To substantiate this notion, we analyzed our Pol2 ChIP-seq experiments in more detail and observed a difference in the Pol2 pileup around the integration site based on the direction of the insert (Fig. 5a). The Ef1a insert in the forward direction had a sharper Pol2 peak downstream of the landing pad, suggesting that in this orientation transcription likely terminated within 1.5 kb after the polyA site (Fig. 7g). In contrast, the reverse insert had a more dispersed signal, with a major peak ~ 3.5 kb after the polyA signal and detectable Pol2 enrichment as far as 5 kb downstream (Fig. 7g). These observations can explain why insertions in the reverse direction had lower mTagBFP2 levels (due to increased read-through and inverse correlation of transcript length and expression^40^) but yielded more competition than those in the forward direction. Indeed, deletion of the polyA signal in the Forward Ef1a reporter – which results in read-through of up to 5kb – attenuates eGFP signal to a degree comparable with the reverse Ef1a reporter containing the intact polyA (Fig. 7d, Extended Data Fig. 7). Altogether, our data suggest that transcript level and length are major determinants of promoter competition.

## DISCUSSION

In this work, by systematically introducing *de novo* promoters to the *Sox2* locus, we revealed the importance of promoter position, strength, and the associated transcriptional output as the main determinants of promoter competition. In addition to shedding light on new aspects of promoter competition, our work agrees with previous suggestions that a simple enhancer resource sharing model is insufficient to explain promoter competition. Going back to the a-globin locus, the *de novo* promoter only impacts endogenous globin gene expression when it is in-between the globin genes and the locus control region (LCR). Placing the same promoter upstream of the LCR, does not disrupt gene expression, even though it remains within the same TAD^12^. Similarly, we do not observe promoter competition when the insert is in the downstream landing pad, but only when it is in between the *Sox2* promoter and its SE, despite promoters in both locations receiving comparable regulatory input from the SE. This demonstrates that the *Sox2* SE can support the transcription of multiple promoters. Moreover, it establishes that promoter competition is not caused by the new promoter sequestering resources from the cognate promoter. Consistent with these observations, other studies show that gene promoters do not structurally compete for interactions with enhancers but rather, form a regulatory hub structure in which multiple promoters can simultaneously contact and be activated by an enhancer^41,42^. While genomic position of the promoter is clearly relevant, the location of the competing promoter between the enhancer and the dampened promoter is not an absolute requirement for competition. This is suggested by observations at the b-globin locus, where the activation of the adult b-globin promoters, located downstream of both the LCR and the fetal globin promoter, dampens expression of the latter^7^. We propose that the position of the competing promoter is important inasmuch it affects formation of the regulatory hub structure. In that context, the competition between promoters within the same regulatory hub is likely mutual.

We note that quantitatively, the effects of promoter competition observed in our system are subtle, with the strongest competing promoter reducing the transcription of the endogenous gene by about 2-fold. However, gene dosage effects of this magnitude may have profound consequences on biology. For example, haploinsufficiency – where the gene dosage is reduced by half – is a common cause of human disease^43^. Moreover, scans of copy number variation in nearly a million of human genomes predicted that 2,987 human genes are haploinsufficient and 1,559 are triplosensitive^44^. Such widespread dosage sensitivity implies that at endogenous loci, the activity of cis-regulatory elements must be calibrated to account for promoter competition or alternatively, promoter competition must be actively counteracted. Both are likely true, and in support of the counteracting mechanism, we observed HUSH-mediated silencing of the ectopic promoters at the *Sox2* locus. Considering that HUSH-mediated silencing is co-transcriptional and that its canonical targets are retroviruses or transposable elements with transcription potential^17,20,22,45^, it follows that HUSH provides the first-defense mechanism that protects from deleterious effects of promoter competition associated with the *de novo* promoter insertions resulting from transposon insertions and other genomic rearrangements. However, HUSH silencing does not solve the conundrum of how the competition among endogenous promoters that need to remain active within the same regulatory domain is resolved. Our data shows that promoter strength and position govern competition, and thus one potential solution for avoiding promoter competition is placing the ubiquitously active promoters (which typically are ‘housekeeping’ promoters) away from the centers of regulatory domains. Indeed we know that boundaries of topologically associated domains (TADs) are enriched for highly and ubiquitously transcribed promoters^46^. In contrast, the centers of these TADs are depleted of such promoters^47^. We propose that this genome organization may have evolved to avoid competition from the strongest housekeeping promoters within the regulatory domains, while also reinforcing the boundary function. Consistent with the latter notion, in some contexts promoters^47,48^ or simple transcribing units^49^ have been shown to provide an enhancer-blocking function, and this can be interpreted in the light of the transcription-driven promoter competition described here.

Our work suggests the importance of transcript levels and length as key determinants of competition strength. However, a molecular mechanism underlying this phenomenon is still elusive. One possible explanation is the role of RNA as a regulator of condensates^50^. Super enhancers, such as the one studied in here, were shown to nucleate transcriptional condensates by recruiting high amounts of activating transcription factors and cofactors^51^. Super-resolution imaging has previously established this to be the case at the *Sox2* locus, where transcriptional condensates enhanced bursting of the Sox2 promoter when in close proximity^52^. On the other hand, it has been posited that while low levels of RNA might help transcriptional condensate formation, high levels of RNA, associated with bursting and elongation, dissolve said condensates^53,54^. As such, in our system we observed that the more abundant and longer the transcript driven by the integrated promoter, the greater the interference with endogenous *Sox2* transcription. While future studies should more directly address the role of RNA in promoter competition, the results described here lay the groundwork for understanding how presence of multiple promoters within complex regulatory domains may influence gene expression.

## METHODS

### Cell Culture

F123 mESCs were a gift from Bing Ren’s laboratory at UCSD^18^ and were cultured as previously described.^55^ In brief, cells were grown in monolayer on tissue culture plates pretreated with 7.5 ug/ml poly-L-ornithine (Sigma, P4638) in PBS and 5 ug/ml laminin (Gibco, 23017015) in PBS consecutively for at least 1 hour at 37°C. Cells were maintained in serum-free 2i + LIF media (500mL DMEM/F-12 (Gibco, 11320082), 1x Gem21 Neuroplex w/o Vitamin A (Gemini Bio, 400-161-010), 1x N2 Neuroplex (Gemini Bio, 400-163-005), 2.5g BSA (Gemini Bio, 700-104P), 1xMEM NEAA (Thermo, 11140050), 1xSodium pyruvate (Thermo, 11360070), and 1xAnti-Anti (Sigma, A5955) containing MEK inhibitor PD0325907 (0.8 uM, Selleck, S1036), GSK3b inhibitor CHIR99021 (3.3 mM, Selleck, S2924), and leukemia inhibitory factor (LIF, produced in-house). Media was changed every day, cells were passaged every 2-3 days when 80-90% confluent using 0.25% Trypsin-EDTA (Gibco, 25200072) to release cells from the plate. Dissociation reaction was quenched with quenching media (500mL DMEM/F-12, 1x Anti-Anti(Sigma), 10% FBS (Gemini Bio, 100-106)). Cells were re-plated on PLO/Laminin plates at 1:6-1:20 dilutions.

### Donor plasmid cloning for RMCE

The donor vector was adapted from pGL3-Basic plasmid (Promega, E1751). First, pGL3 was modified to remove luciferase and SV40 polyA signal. Two ultramers containing FRT and FRT3 (IDT), and a gene-fragment with a multiple cloning site (MCS), followed by mTagBFP2 with a mouse b-globin intron and an SV40 polyA site (Twist Biosciences) were ordered with matching restriction sites. The empty pGL3 backbone was re-ligated with these three components to create parental donor plasmids. To allow for inserts in different orientations, two versions of the donor plasmid were made with the same strategy (for pDonor-For, FRT-mTagBFP2-FRT3, for pDonor-Rev, FRT3-mTagBFP2-FRT). 1kb fragment of promoters were amplified from mouse genomic DNA with nested PCR using Platinum SuperFi II polymerase (Thermo, 12361010) and neutral fragment was amplified directly from DH5-alpha cells (NEB, C2987H) with overhangs matching the MCS. Ef1a (Addgene #55632) and CAG (Addgene #161974) were taken from published plasmids. For Ef1a allelic series and Rpl13a mutant experiments, the fragments were ordered from Twist. For noSV40 PolyA experiments parental donor plasmids were modified to remove the SV40 polyA with a 20bp neutral fragment, followed by Ef1a promoter insertion into the MCS. All plasmids were verified with analytical digest, followed by whole plasmid sequencing (Plasmidsaurus). All primers used and promoter sequences are listed in Supplementary Table 1.

### Landing pad – RMCE cell lines

Parental landing pad lines (LP_bw or LP_ds) were co-transfected with a Flippase expression plasmid (Addgene #89574) and donor plasmid with promoter at 1:2 molar ratio using Lipofectamine 2000 (Invitrogen, 11668019) according to manufacturer protocol. Cells were allowed to recover for 7-10 days after transfection and passaged onto 10cm plates with media containing 0.5uM ganciclovir (Sigma, SML2346). Surviving isolated clones were manually picked and transferred to a 48-well plate. After another week in culture, cells were passaged for maintenance and genomic DNA collection with Quick Extract (Lucigen, QE09050). Clones were genotyped with primers specific to forward or reverse inserts using OneTaq Master Mix with GC buffer (NEB, M0489L). At least three independent clones were kept for each promoter insert for subsequent flow cytometry analysis.

### Generation of HUSH Knockout Cell lines

To stably express targeting guides and Cas9, px459(Addgene, 62988) plasmid was cloned into a PiggyBac backbone to generate pb459. Two guides targeting Mpp8 and two non-targeting^22^ control guides were cloned into pb459 using golden gate assembly. One clone from each promoter insert was selected and transfected with either two Mpp8 targeting or two non-targeting guide plasmids, alongside Supertransposase (SBI, PB210PA-1) in 1:1:1 equimolar ratio using lipofectamine 2000 according to manufacturer recommendations. Next day populations were passaged onto 2i+LIF media containing 1ug/ml puromycin (Invivogen, ant-pr-1). The parental clones were grown alongside the Mpp8 deletion and non-targeting controls and assayed together in flow cytometry experiments.

To establish clonal Mpp8 deletion lines, the same two Mpp8 targeting guides were cloned into px459. Ef1a and Neutral insert lines were transfected with px459 plasmids. Next day populations were passaged onto 2i+LIF media containing 1ug/ml puromycin. After 3 days of selection, 1000-2000 surviving cells were plated on a 10cm plate coated with 5ug/ml fibronectin (Sigma, FC01010MG) in PBS. After 1 week of culture, individual clones were picked, genotyped. Selected clones were validated with western blot for Mpp8 knockout. All guides and primers used for genotyping are listed in Supplementary Table 2.

### Generation of SE deletion lines

To delete the SE in an allele specific manner, two guides with PAM sequences overlapping CAST SNPs were chosen. Two SE targeting guides were cloned into px459 and Ef1a insert lines were transfected with these plasmids. Next day populations were passaged onto 2i+LIF media containing 1ug/ml puromycin. After 3 days of selection, surviving cells were sorted in Sony MA900 cell sorter (housed in Stanford Stem Cell FACS Facility) for loss of GFP and maintenance of mCherry signal. 1000-2000 sorted cells were plated on a 10cm plate coated with 5ug/ml fibronectin in PBS. After 1 week of culture, individual clones were picked and later genotyped. At least three independent clones were kept for each SE deletion for subsequent flow cytometry analysis. All guides and primers used for genotyping are listed in Supplementary Table 2.

### Generation of Rad21-dTag lines

A guide^56^ targeting the C-terminal of Rad21 was cloned into px459. A donor plasmid with ~1.1kb Rad21 homology arms flanking FKBP12^F36V^-Flag-T2a-Puro was generated with Gibson assembly. Ef1a insertion lines were co-transfected with donor and px459 plasmid at a 1:1 molar ratio. Next day populations were passaged onto 2i+LIF media containing 1ug/ml puromycin. After 3 days of selection, 1000-2000 surviving cells were plated on a 10cm plate coated with 5ug/ml fibronectin in PBS and kept in puromycin containing media. After 1 week of culture, individual clones were picked and genotyped. Successful clones were confirmed with DMSO vs 500nM dTag treatment for 6 hrs followed by western blot to assure maintenance of tagged Rad21 levels and proper degradation. All guides and primers used for genotyping are listed in Supplementary Table 2.

### CARGO Array and Cell Population Generation

CARGO 6-mers were generated as described previously^37,57^. Briefly, CARGO constant region was digested from pGEMT-hU6-SL. Custom oligos were annealed and phosphorylated. Each annealed oligo independently was combined with the digested CARGO constant region to generate minicircles. Minicircles were then treated with Plasmid-safe exonuclease to remove unligated fragments according to the manufacturer’s protocol. Minicircles were then combined and cleaned using Zymo DNA Clean & Concentrator-5 (Zymo, D4014) kit. Separately CARGO plasmid backbone was generated from a modified version of pK335 (Addgene, #227505) plasmid was generated with no florescence reporter, pK335-NoColor with restriction digest. CARGO array was then generated by combining the minicircles and the CARGO plasmid backbone at a 3:1 molar ratio in a golden gate reaction. Assembly reaction was then treated with Plasmid-safe exonuclease per the manufacturer’s protocol. 1uL of this reaction was used to transform 25uL NEB Stable competent E.coli (NEB, C3040H). Successful preps were confirmed with full plasmid sequencing (Plasmidsaurus). Clonal Ef1a or neutral insert lines were transfected with PhiC Integrase (SBI, FC200PA-1 and either BFP Gene targeting or non-targeting CARGO in equimolar ratio. Next day, cells were passaged onto 2i+LIF media containing 200ug/ml G418 (Invivogen, ant-gn-1). After 7 days, populations were transfected with pB-dCas9-Puro plasmid. Next day, cells were passaged onto 2i+LIF media containing 200ug/ml G418 and 1ug/ml puromycin. A no-dCas9 expression control was also performed but all comparisons are between Targeting and Non-targeting populations with dCas9 expression. Cells were then grown and expanded for 10 days in double selection media. On 11^th^ day, a portion of cells were crosslinked for ChIP and the remainder were processed with flow cytometry. Independent populations were generated as described each time for 3 biological replicates. All guide RNA sequences and custom oligos for CARGO synthesis are provided in Supplementary Table 2.

### Flow cytometry data acquisition and analysis

For promoter screens such as ones depicted in Fig1E, Fig 2D, Supp 2B, Supp 3B, cells were grown on 48-well plates. Cells were detached from plates with trypsin and resuspended with 2i+LIF media containing 5% serum (Gemini, 100-525). Resuspended cells were mixed thoroughly and transferred to a U-bottom 96 well plate. Then, cells were analyzed in a LSR Fortessa analyzer (BD) housed in Stanford Stem Cell FACS facility, in High Throughput Sampler mode. eGFP, mCherry and mTagBFP2 signal were recorded for at least 10000 cells for each clone, with hardware compensation. For experiments with less than 30-40 samples, cells grown in 6 or 12-well plates were released with trypsin, quenched and spun down. The pellet was resuspended with cold FACS buffer (1xPBS, 5% FBS), cells were strained through 0.35uM mesh for subsequent analyses in LSR Fortessa, in tube mode and at least 50000 events were collected.

The FCS files were processed in FlowJo (v10.10.0) and gated for singlets (consecutively gated for SSC.A vs FSC.A, FSC.H vs FSC.A, FSC.W vs FSC.A, SSC.H vs SSC.A, SSC.W vs SSC.A). The linear values from gated populations were exported as csv files and further analyzed in R (v4.3.3). The files were imported into R and linear regression were performed to account for SSC and mCherry contribution (lm(log2(Flurophore) ~ log2(SSC)+ log2(mCherry)), with the exception of SE deletion experiments, which were only accounted for SSC, because wild type cells with no fluorophores were used as control. Residuals from this linear regression were used in downstream analyses and plotting.

### Luciferase reporter assays

Ef1a mutant promoters were cloned into pGL3-Basic (Promega, E1751) using restriction digestion. Two distinct preps for each mutant promoter were cleaned with Plasmid-Safe ATP-Dependent DNase (Lucigen, E3101K). Transfections were performed in 24-well plates, with each well receiving 10ng of pGL3 plasmid, 0.5ng of control pRL firefly renilla plasmid (Promega E2261), 89.5 mL carrier DNA (circularized pGEMT plasmid) and 0.25ul of Lipofectamine in 50ul Optimem (Gibco 31985070). 24 hours after transfection, cells were assayed with Dual-Luciferase Reporter Assay System (Promega, E1960) according to manufacturer’s recommendation. Briefly, cells were washed in PBS, and lysed in 100 uL 1X passive lysis buffer (in PBS) for 10 min. 20 uL lysate was then transferred to an opaque flat-bottomed plate for reading with a luminometer (Promega, GloMax). The automated injector added 100 uL LARII reagent and the well was read using the following parameters: 2 s delay, 10 s integration. 100 uL Stop-and-Glow reagent was then injected into the well and read using the same parameters. Luciferase assays were repeated twice, using biological duplicates (two distinct preps of same promoter vector), transfected in duplicate; empty vector and SV40 promoter were included in each experiment as controls.

### Total cell lysis and Western Blot

Cells were washed once with PBS before lysing on-plate in ice (1 mL per 10 cm plate) with ice cold RIPA buffer (50 mM Tris-HCl pH 7.4, 150 mM NaCl, 1 mM NP40, 0.5% sodium deoxycholate, and 0.1% SDS) with 1x cOmplete EDTA-free protease inhibitor cocktail (Roche, 11873580001) and 1mM PMSF. The lysate was centrifuged for 10 minutes at max speed (>15,000 x g) at 4°C. The supernatant was moved to a new tube and protein concentration was quantified using the Pierce BCA protein kit (Thermo Scientific, 23227) and normalized. Samples were then denatured by addition of 1x NuPAGE LDS Sample Buffer (Invitrogen, NP0007) and 1.25% b-mercaptoethanol and subsequently heating to 70° C for 10 min. Samples were loaded to 4-20% Novex Tris-glycine gels (Invitrogen) and run at 100V for 30min, then 180V for 40min in Tris-glycine buffer (25 mM Tris and 192 mM glycine) with 0.1% SDS. Gels were transferred onto nitrocellulose membranes (GE Healthcare, 10600003) for 1 h at 400 mA in Tris-glycine buffer with 20% methanol. The membrane was stained with 0.1% Ponceau S in 3% trichloroacetic acid, then blocked with 5% milk and 1% BSA in PBS with 0.1% Tween-20 (PBST) for 30 min at room temperature and then incubated with primary antibody overnight at 4°C followed by horseradish peroxidase (HRP)-conjugated secondary antibody incubation for 1 h at room temperature, with 4 washes of PBST after each antibody incubation. Chemiluminescence was performed with Amersham ECL Prime reagent (Cytiva, RPN2232) and imaged with an Amersham ImageQuant 800 (Amersham). Membranes were then incubated for 1 h at RT with HRP-conjugated loading control antibodies, washed and imaged again. Antibodies used include Mpp8 (Proteintech, 16796-1-AP), Rad21 (Abcam, ab992), b-actin-HRP (Abcam, ab49900), Hsp90-HRP (Cell Signaling, 79641S), Goat-Rabbit IgG (H+L) HRP (ProteinTech, SA00001-2).

### ChIP-qPCR & ChIP-Seq

10-30 million sub-confluent cells were grown on 6 cm (for H3K9me3) or 10 cm (for Pol2, Rad21, dCas9) plates, washed with 1x PBS and fixed with freshly prepared 1% methanol-free formaldehyde in PBS for 10 minutes at room temperature with nutation. Glycine was added to a final concentration of 125 mM to quench for an additional 10 minutes with nutation. The quenched formaldehyde solution was removed, and cross-linked cells were scraped from plate in ice cold PBS with 0.001% Triton-X100. Cells were pelleted at 1350xg for 5 minutes at 4°C, washed with ice cold PBS, and pelleted as before. All liquid was removed, pellets were snap-frozen in liquid nitrogen and stored at −80°C until further processing.

On the day of the immunoprecipitation, pellets were thawed on ice for 30 minutes and resuspended in 5 mL LB1 (50 mM HEPES-KOH pH 7.5, 140 mM NaCl, 1 mM EDTA, 10% glycerol, 0.5% NP-40, 0.25% Triton X-100, with 1X Roche cOmplete Protease Inhibitor Cocktail and 1mM PMSF) , rotated end over end at 4°C for 5 minutes, then pelleted at 1350xg for 5 minutes at 4°C. The pellet then was resuspended in 5mL LB2 (10 mM Tris-HCl pH 8.0, 200 mM NaCl, 1 mM EDTA, 0.5 mM EGTA, with 1X Roche cOmplete Protease Inhibitor Cocktail and 1mM PMSF) and again rotated end over end at 4°C for 5 minutes, then pelleted at 1350xg for 5 minutes at 4°C. Lysates were then resuspended in ice cold LB3 (10mM Tris-HCl, pH 8.0, 100mM NaCl, 1mM EDTA, 0.5 EGTA, 0.1% Na-Deoxycholate, 0.5% N-laurylsarcosine with 1x Roche cOmplete Protease Inhibitor Cocktail and 1mM PMSF) and transferred to appropriate tubes and incubated on ice for 10 minutes. For samples processed for ChIP-seq, 1ml LB3 was used, and sample was transferred to Covaris AFA tubes and sonicated for 10 minutes at peak power = 140, duty factor = 10, and cycles per burst = 20 in the E220 evolution Covaris. For samples that were being processed for ChIP-qPCR 0.3ml LB3 was used, and sample was transferred to Diagenode TPX tubes. ChIP-qPCR samples were sonicated for 8 cycles of 30s on/ 30s off on high power using Bioruptor Plus (Diagenode), additional cycles added until chromatin peak was observed around 500bp. To quantify total chromatin amount, and determine whether additional cycles of sonication were necessary, 5 ul out of 300ul (or 5 out of 1ml) were taken out and the rest of the sample kept on ice. To each 5uL sample, 195uL elution buffer (1% SDS, 0.1M NaHCO3), 2ul 5M NaCl, and 1ul RNaseA (0.2mg/ml final) was added. Samples were incubated at 65°C for 1hour with shaking. 1ul ProteinaseK (0.2mg/ml final) was added and samples were incubated at 65°C for an additional 1hour with shaking. DNA was then extracted using Zymo ChIP Clean & Concentrator-5 (Zymo, D5205) kit according to the manufacturer’s protocol. Portion of eluted DNA was run on an agarose gel to see sheared chromatin size and if desired peak at 500bp was achieved, DNA was quantified with Qubit DS High Sensitivity Kit (Thermo, Q33231). If chromatin peak was above 500 bp, additional cycles of sonication performed and then validation and quantification step repeated. The remaining sonicated lysate was diluted to 1mL in LB3 and spun at max speed (>15000xg) for 10 minutes at 4°C. Supernatant was transferred to a fresh DNA LoBind tube and Triton-X100 was added to a final concentration of 1%. Based on Qubit measurements, chromatin was normalized and 5% input samples were reserved and stored at −20°C. For H3K9me3 10ug, for Rad21 ChIP-seq samples 20ug, for Pol2 ChIP-seq samples 15ug, for Rad21, Pol2 and Cas9 ChIP-qPCR samples 20ug chromatin was used. For each IP, 5ug antibody was added to 1ml soluble chromatin sample. Then samples were rotated vertically overnight (12-16hr) at 4°C. Antibodies used include H3k9me3(Abcam, ab8898), Rad21 (Abcam, ab992), RNA Pol 2 (Biolegend, 664906), Cas9 (Active Motif, 61757).

The next day, 100uL Protein G Dynabeads (Thermo, 10004D) per ChIP sample were washed 3 times with ice cold blocking buffer (0.5% BSA(w/v)(Sigma, A3294) in PBS) then ChIPs were added to beads and incubated with vertical rotation for 4-6 hours at 4°C. ChIPs were then washed 5 times with RIPA buffer(50mM HEPES-KOH, pH 7.5, 500mM LiCl, 1mM EDTA, 1% NP-40, 0.7% Na-Deoxycholate), once with TE + 50mM NaCL (50mM Tris-HCl, pH 8.0, 10mM EDTA, 50mM NaCl), then eluted from the bead in 200uL elution buffer(1% SDS, 0.1M NaHCO3) at 65°C for 30min and eluate was transferred to a new DNA LoBind tube. To reverse cross-link and degrade RNA, 8uL 5mM NaCl and 2uL RNaseA (0.2mg/ml final) was added to eluate and to thawed input samples that were diluted to 200uL in elution buffer. All samples and inputs were incubated overnight (12-16hr) at 65°C with shaking. ProteinaseK (0.2mg/ml final) was added to samples and inputs, which were incubated for another 2 hours at 55°C with shaking. For ChIP-seq, DNA was extracted with phenol chloroform extraction and for ChIP-qPCR samples were processed using Zymo Clean& Concentrator-5 kit. DNA was then stored at −20°C.

For ChIP-seq, libraries were prepared using the NEBNext Ultra II DNA kit (NEB, E7645S) using up to 50 ng of input or ChIP DNA, with ~4-8 cycles of amplification, with no pre-PCR size selection but a post-PCR double-sided 0.5x/0.9x Ampure XP bead clean-up. Final quantification and QC were assessed by Qubit and Tapestation (Agilent), respectively, and libraries were pooled for sequencing on NovaSeq X Plus (Illumina) platform.

For ChIP-qPCR, DNA samples were diluted by a factor of 5. Master mixes containing 5uL/reaction Sensifast SYBR No-Rox 2x mix (Meridian Bioline, 98020), 0.25ul 10mM primer mix, and 0.75uL water were prepared and kept on ice. 6uL of appropriate qPCR master mix was added to each well of a 384 well plate followed by 4uL of diluted cDNA. All samples were run in technical triplicate in a LightCycler 480 (Roche).

### ChIP-seq Analysis

An N-masked genome and a SNP file for Cast_EiJ and 129/Sv were generated using SNP annotation VCF file from the Mouse Genomes Project (Release V8, 12/2021) with the SNPSplit (v0.6.0) in dual strain mode. The reads were trimmed using skewer (v0.2.2) and aligned to the N-masked mouse genome using bowtie2(v2.5.1). After alignment the reads were filtered for quality score and PCR duplicates were removed. Then, the reads were assigned to either Cast or 129 allele with SNPSplit. For visualization, RPGC normalized allele specific bigwig files were generated using deeptools (v3.5.1) bamCoverage.

### Analysis of Public ChIP-seq data for promoter features heatmap

Public datasets (SRAs listed in Supplementary Table 1) were downloaded with sratoolkit (v3.0.7). Adaptors were trimmed using Fastp (v0.21.0) and Fastq files were aligned with Bowtie2 (v2.5.1) to mm39. The reads were filtered for quality score and PCR duplicates were removed from only paired-end datasets. Bam files were normalized and converted to bigwig files with bamCoverage from deeptools (v3.5.1) using RPGC normalization. All bigwig files were then analyzed together with multiBigwigSummary from deeptools to create a coverage matrix over 1kb regions of endogenous promoters. The coverage values were log transformed. Scaled values were plotted as a heatmap of relative enrichment.

### RT-qPCR

Cells were grown in 12-well plates. For Rad21-dTag experiments, cells were treated with 500nM of dTagv-1 or DMSO for 6 hours. For each experiment, more than 5 biological replicates (clones) were collected and assayed twice. Prior to collection, cells were washed with PBS at RT, then 400uL Trizol reagent (Invitrogen, 15596026) was added to each well. Plates were nutated for 5 minutes at RT and samples were then transferred to RNase free tubes and stored at −80°C until further processing.

To extract RNA, samples in Trizol were thawed on ice, then total RNA was extracted using Zymo Direct-zol (Zymo, R2052) kit and cDNA was prepared from 2ug RNA using High Capacity cDNA Reverse Transcription Kit (Invitrogen, 4368813) according to manufacturer’s protocol. cDNA samples were diluted by a factor of 20 prior to RT-qPCR. Master mixes containing 5uL/reaction Sensifast SYBR No-Rox 2x mix (Meridian Bioline, 98020), 0.25ul 10mM primer mix, and 0.75uL water were prepared and kept on ice. 6uL of appropriate qPCR master mix was added to each well of a 384 well plate, followed by 4uL of diluted cDNA. All samples were run in technical triplicate using a LightCycler 480 (Roche).

### Analysis of qPCR data

All qPCR primers were validated prior to use in all experiments with the requirement to generate a single PCR product as determined by melt curve analysis and to have an efficiency of 1.8-2.0. Standard curves for all validated primer sets were obtained and used to convert Cp values into relative concentrations. Primers are provided in Supplementary Table 2.

All individual qPCR replicates are the average of technical triplicates. All ChIP-qPCR samples are quantified as percent input recovery. For RT-qPCR sample concentrations were normalized to a housekeeping gene, Rpl13a, then normalized by expression for a control line Neutral insert for transcript length experiments that was normalized by Rpl13a. For Rad21 dTag experiments, Gapdh was used as a housekeeping control, and housekeeping normalized samples were then normalized by mCherry expression in their respective DMSO control counterpart. For experiments in Fig7c and Supp7, sample concentrations were only normalized to Rpl13a and not normalized further to a cell line, to display absolute levels of transcript, not relative levels.

## Extended Data

## Data Availability

Genomic datasets have been deposited at GEO and will be publicly available as of the date of publication. Plasmids generated in this study will be deposited at Addgene and released upon publication. All processed data are available in supplementary files. Raw data and cell lines are available upon request from wysocka@stanford.edu
